# Newborn Intravenous Injection of Liposomal CRISPR/Cas9 Complex Has No Incidence of Off-Targets or Tumors in Mice

**DOI:** 10.3390/pharmaceutics17050656

**Published:** 2025-05-17

**Authors:** Vinícius Monteagudo, Larissa Cristina Barbosa Flores, Melaine Lopes, Flavia Nathiely Silveira Fachel, Giselle Martins, Marina Siebert, Willian da Silva Carniel, Tuane Nerissa Alves Garcez, Helder Ferreira Teixeira, Ursula Matte, Roberto Giugliani, Guilherme Baldo, Édina Poletto, Roselena Silvestri Schuh

**Affiliations:** 1 Graduate Program in Pharmaceutical Sciences, Universidade Federal do Rio Grande do Sul, Avenida Ipiranga, 2752/lab 606, Porto Alegre 90610-000, RS, Brazil; vmbarros@hcpa.edu.br (V.M.); larissacbflores12@gmail.com (L.C.B.F.); mmslopes@hcpa.edu.br (M.L.); flavia.fachel@ufrgs.br (F.N.S.F.); carnielwillian@gmail.com (W.d.S.C.); helder.teixeira@ufrgs.br (H.F.T.); 2 Cells, Tissues, and Genes Research Group, Experimental Research Center, Hospital de Clínicas de Porto Alegre, Porto Alegre 90035-903, RS, Brazil; dra.marinasiebert@gmail.com (M.S.); tgarcez@hcpa.edu.br (T.N.A.G.); umatte@hcpa.edu.br (U.M.); gbaldo@hcpa.edu.br (G.B.); 3 Graduate Program in Genetics and Molecular Biology, Institute of Biosciences, Universidade Federal do Rio Grande do Sul, Porto Alegre 90010-150, RS, Brazil; 4 Department of Pediatrics, Stanford University, Stanford, CA 94305, USA; edinapoletto@gmail.com

**Keywords:** CRISPR/Cas9, gene editing, liposome, off-target, tumor

## Abstract

**Background**: Genome editing at specific loci is an innovative therapeutic approach; however, it faces many challenges, so optimizing delivery vectors is essential to enhance the safety and efficacy of the CRISPR/Cas9 system. This study investigated whether the hydrodynamic administration of liposomal CRISPR/Cas9 complexes (LCs) in newborn mice induces off-target events or tumors. **Methods**: Liposomes were obtained through microfluidization. The CRISPR/Cas9 plasmid and a donor plasmid containing the *Idua* cDNA (alpha-L-iduronidase enzyme) were incorporated by adsorption, and complexes (LCs) were characterized regarding physicochemical properties. C57BL/6 newborn mice were divided in two groups, one received the complexes through hydrodynamic intravenous injection (n = 15) and the other was used as control (n = 15). After 21 months, mice were euthanized and organs were analyzed regarding histological characteristics. Lungs and liver were analyzed by qPCR searching for potential off-target sites in chromosomes 2, 5, 11, and 17 and on-target site in chromosome 6, identified by COSMID. Sequences were analyzed using an ICE tool for indels detection. **Results**: LCs exhibited 136 nm mean vesicle diameter with PDI below 0.15 and a zeta potential around +43 mV. Immediate biodistribution was predominant in the lungs and liver. There was no significant increase in tumor induction (20% in LCs vs. 33% in control). Molecular analyses indicated 0% off-target effects and around 3% on-target events. **Conclusions**: We conclude that this set of experiments demonstrates the potential of the chosen gRNA sequence to perform safe gene editing at the murine *ROSA26* locus, corroborating the safety of the CRISPR/Cas9 gene editing platform.

## 1. Introduction

Gene therapy has been used since the last century as a hopeful alternative for treating various genetic diseases. New gene therapy approaches are emerging and increasingly becoming a more viable option for the treatment of pathologies. In early 2013, genomic editing took a significant leap with the discovery of the CRISPR system (Clustered Regularly Interspaced Short Palindromic Repeats), which was initially identified as an adaptive defense mechanism in bacteria against invading bacteriophages. Currently, this technology has been adapted to mammals and is being widely used as a genomic editing tool. The CRISPR/Cas9 platform consists of a Cas9 protein, which acts as an endonuclease guided by a guide RNA (gRNA) complementary to the target genomic sequence. It works in conjunction with a CRISPR RNA (crRNA), with its sequence homologous to the target site, and the trans-activating CRISPR RNA (tracrRNA), both of which are essential for directing the Cas9 nuclease to the desired site. Subsequently, the Cas9–sgRNA complex performs the cut at the target genomic locus. When an additional donor sequence is provided with the system, homologous recombination can occur at the cleavage site of the cellular genome [1,2]. However, the efficiency of the CRISPR/Cas9 system depends on overcoming some barriers, such as targeting to the specific cell or tissue, cellular penetrability, patient’s immune response, and off-target events. Therefore, it is essential to focus on improving delivery strategies in gene editing to increase both the efficacy and safety of treatments.

Regarding cellular internalization, mainly viral and non-viral vectors can be used. Viruses have great efficiency in penetrating host cells and replicating, with viral particles being modified to remove the genes responsible for replication, thereby allowing for safer integration of the therapeutic system. Despite being the most used, viral vectors have limitations related to the packaging of large nucleic acid sequences, immunogenicity, and the durability of Cas protein expression, which can lead to off-target events [3]. On the other hand, non-viral vectors have a greater capacity to accommodate long sequences of nucleic acids and proteins such as Cas9 mRNA, sgRNA, donor DNA, and RNPs. They are also more biocompatible, show lower immunogenicity, and demonstrate lower toxicity and production costs. However, these vectors have lower transfection efficiency within the cell [4,5].

Among the biomaterials used in non-viral vectors to transport nucleic acids, cationic lipids and polymers are highlighted. These materials form complexes with DNA, maintaining a residual positive charge to bind to these polyanions. In this way, liposomes composed of cationic lipids can be produced. The nucleic acids are adsorbed to the surface of the vesicles or inside the aqueous core by association with the cationic lipid, being protected from degradation and transported into the cell through this carrier [4,6].

In this sense, to assess the safety of gene editing with the CRISPR/Cas9 system, the hydrodynamic administration of liposomal vectors complexed with the plasmids of the CRISPR/Cas9 system and a donor of the complete cDNA of *Idua* gene in newborn mice was performed. Our research group evaluated the complexes for the treatment of MPS I mice (mucopolysaccharidosis type I) in order to correct the defect in the gene of the alpha-L-iduronidase enzyme (IDUA, EC 3.2.1.76), responsible for the catabolism of the glycosaminoglycans (GAGs) dermatan and heparan sulfate [7]. Experimental treatment using the complexes demonstrated increased enzyme activity in the previous work, reaching about 5% of normal mice serum and tissue *Idua* activity levels [7,8].

Considering the positive results achieved in previous studies and aiming to evaluate the safety of the treatment, we intent to investigate the potential tumor induction from the injection of these complexes in normal newborn mice as well as its potential to produce off-target effects derived from Cas9 cleavage. Given that the CRISPR system utilizes configurations of gRNAs, which can occasionally cause mutations in unintended genomic loci, off-target genome editing may occur. This can lead to various outcomes, including oncogene activation due to deleterious effects, tumor induction, immune response, and cytotoxicity. Therefore, studies are necessary to identify the presence of off-target mutations [9].

Finally, in view of the CRISPR/Cas9 efficiency previously demonstrated and based on the need to investigate this promising approach, the safety and tumor induction of the complexes were assessed in vivo to evaluate the potential to produce off-target effects, awaiting that this treatment can, in the near future, be used as therapy in patients affected by MPS I.

## 2. Materials and Methods

### 2.1. Vectors

The PrecisionX CRISPR/Cas9 SmartNuclease™ system (System Biosciences, Palo Alto, CA, USA) was used for in vivo genomic editing experiments, and the target sequence for cleavage by the Cas9, 5′ggattctcccaggcccaggg3′, was selected at the *ROSA26* locus of the mouse genome and was inserted in the plasmid vector [10].

For homologous recombination, a vector containing the *Idua* cDNA customized by System Biosciences (USA) was used. The construct contains the mouse *Idua* cDNA sequence regulated by an EF promoter and two homologous regions (approximately 1 Kb each) to the *ROSA26* locus of mice, in the region that Cas9 recognizes and cleaves.

### 2.2. Preparation of Formulations and Complexes

Liposomal complexes (LCs) and fluorescent liposomal complexes with NBD-PE (N-(7-Nitrobenz-2-Oxa-1,3-Diazol-4-yl)-1,2-Dihexadecanoyl-snGlycero-3-Phosphoethanolamine, Triethylammonium Salt) fluorescent labeled phospholipid (Thermo Fisher Scientific, Waltham, MA, USA) were prepared by adsorption of both (CRISPR/Cas9 *ROSA26* and donor *Idua ROSA26*) plasmids onto blank liposomes at a +4/−1 charge ratio, as previously described [7].

### 2.3. Characterization of the Complexes

The droplet size and polydispersity index (PDI) were determined by photon correlation spectroscopy at 25 °C after appropriate dilution of samples in water. The ζ-potential was determined by electrophoretic mobility at 25 °C after appropriate dilution with 1 mM NaCl solution. The measurements were performed using Zetasizer Nano-ZS90^®^ (Malvern Instruments, Malvern, UK) equipment.

Nucleic acids complexation was verified by agarose gel electrophoresis. The complexes were electrophoresed in 1% agarose gel stained with SYBR Gold Nucleic Acid Gel Stain (Invitrogen, Waltham, MA, USA). Naked plasmids were used as control.

Morphology characterization through Cryo-Transmission Electron Microscopy (Cryo-TEM-Thermo Fisher Scientific, Waltham, MA, USA) was also performed. Cryo-TEM grids were prepared using an automated vitrification system (Vitrobot Mark IV, FEI, NED). Specimens were prepared in a controlled environment with the temperature and humidity set to 22 °C and 100%, respectively, which prevented sample evaporation during the preparation. Before the application of the sample, the grids were subjected to a glow discharge treatment using an easiGlow discharge system (Pelco) with 15 mA current for 25 s in air atmosphere to make them hydrophilic. A 3 μL sample droplet was deposited on a 300 mesh lacey carbon-coated copper grid (01895-F, Ted Pella, Redding, CA, USA). Specimens were analyzed in low-dose conditions, with a defocus range of −2 to −4 μm, using a Cryo-TEM Talos F200C instrument operating at 200 kV (Thermo, Waltham, MA, USA). Images were acquired using a Ceta 4k × 4k camera (Thermo, Waltham, MA, USA). Sample preparation and data acquisition were performed at the Electron Microscopy Laboratory (LME)/Brazilian Nanotechnology National Laboratory (LNNano/CNPEM, BRA).

### 2.4. Animals

This project was approved by the HCPA Ethics Committee on Animal Use for Research under the number #20160482. Newborn C57BL/6 mice (2–3 days old) (n = 30) were used for the experiments and maintained under controlled light and humidity conditions. The treatments consisted of one hydrodynamic injection (10% of body weight, n = 15) of LCs (called only “Treated” group) in the superficial temporal vein of newborn mice. One control group was used (n = 15). During the experiment, mice were weighed weekly. After 21 months, mice were anesthetized with isoflurane prior to cervical dislocation and saturated through the portal vein with a solution containing at least 20 mL of 0.9% NaCl for 3 min to eliminate blood. At the time of euthanasia and tissue harvest, macroscopic analysis of tissues was conducted to inspect for visible tumor masses and other abnormal anatomic findings.

### 2.5. Histological Analyses

Thick blood films were stained with Guiemsa stain for analysis of hematological malignancies (n = 15 per group). Histological slides made from formalin-fixed and paraffin-processed tissues were stained with hematoxylin-eosin staining for analysis by an experienced pathologist. All histological analyses were conducted blindly to the groups.

### 2.6. Immediate Biodistribution of Fluorescent Complexes After Newborn Injection

One experimental group of newborn mice (n = 4 treated and n = 3 untreated control) received one single injection of fluorescent liposomal complexes in the superficial temporal vein, as described above. One minute after treatment, mice were euthanized by decapitation by guillotine with a sharp blade. Blood was collected in EDTA. Brain, lung, liver, spleen, and kidney tissues were removed and mounted on a metal sample holder using Tissue-tek O.C.T.™ (Sakura Fine Technical, Tokyo, Japan). Then, the block was frozen at −80 °C and cut in 30 µm thick slices with a cryostat (Leica CM 1850, Tokyo, Japan). The slices were mounted on a microscope slide and analyzed under a fluorescence microscope (Olympus BX51TF, Tokyo, Japan). The images were taken at 200× magnification. Fluorescence intensity was measured through Image J software version 1.54 (NIH, Bethesda, MD, USA).

Flow cytometry was performed in whole blood after administration of the fluorescent-labeled complexes. The blood collected in EDTA was diluted in HHBS buffer and analyzed for fluorescence intensity levels through Invitrogen Attune™ NXT flow cytometer (Thermo Fisher Scientific, Waltham, MA, USA). First, an FSC/SSC gate was delineated to define a debris-free gate analysis. Fluorescence intensity from samples was then compared by histogram analysis. The comparison was between treated and untreated groups (n = 3 per group). For each sample, 50,000 events were acquired.

### 2.7. On and off Target Analysis

Part of the animals’ tissues was flash frozen immediately after collection and stored at −20 °C. Liver and lung samples were selected for initial analysis, as they were the organs which showed the highest percentage of editing and enzyme production in the previous studies [7]. Total DNA from these tissues was isolated using the PureLink™ Genomic DNA Mini Kit (Thermo Fisher Scientific, Waltham, MA, USA) according to the manufacturer’s recommendations. Possible sites of off-target action of the gRNA 5′ggattctcccaggcccaggg3′ were identified using the COSMID online tool (https://crispr.bme.gatech.edu/ (20 May 2024)) [11]. Five possible sites were identified on chromosomes 2, 5, 11, 17, and 6. To analyze these regions, primers were designed so that each region was amplified by PCR and produced an amplicon of approximately 700 bp. The primers used were:

Chr2: F-tcaactgtttgagccagctcaagg and R-ggctttgcctggctaacagattac;
Chr5: F-acggcaaaggtagcaggcag and R-agcacgcccactacagggtt;
Chr11: F-gtagataaggagctcaggtagcc and R-ctgccccagatgtagtctgaac;
Chr17: F-gaagtgtatggctgccatgtgc and R-gtggagtttggatggccttcg;
Chr6: F-gctctcccaaagtcgctctg and R-ggagcgggagaaatggatatgaag.

The regions of interest were amplified using the enzyme Phusion Green High-Fidelity DNA Polymerase (2 U/µL) (Thermo Fisher Scientific, Waltham, MA, USA), as recommended by the manufacturer. The amplification reactions were performed in a Veriti^®^ 96-Well Thermal Cycler (Applied Biosystems, Waltham, MA, USA) with an initial denaturation step at 98 °C for 3 min, followed by 35 cycles of 98 °C for 10 s, 64 °C for 10 s, and 72 °C for 20 s and finished with a step at 72 °C for 10 min. Amplicons were purified using the Shrimp Alkaline Phosphatase (SAP) and Exonuclease I (Applied Biosystems, Waltham, MA, USA) according to the manufacturer’s instructions. The sequencing of the samples was performed at the Molecular and Protein Analysis Unit (Centro de Pesquisa Experimental, HCPA, BR) using the ABI 3500 Genetic Analyzer equipment with 50 cm capillaries and POP7 polymer (Applied Biosystems, Waltham, MA, USA). PCR products were labeled using 5.0 pmol of forward primers from each region and 1 μL of BigDye Terminator v3.1 Cycle Sequencing reagent Kit (Applied Biosystems, Waltham, MA, USA) in a final volume of 10 μL. The labeling reactions were performed in a Veriti^®^ 96-Well Thermal Cycler (Applied Biosystems, Waltham, MA, USA) with an initial denaturation step at 96 °C for 1 min followed by 35 cycles of 96 °C for 15 s, 50 °C for 15 s, and 60 °C for 4 min. After labeling, the samples were purified by precipitation with the BigDye XTerminator Purification Kit (Applied Biosystems) and electroinjected into the genetic analyzer by Sanger sequencing. The resulting chromatograms were analyzed using the ICE Analysis software version 3 (Synthego, Redwood City, CA, USA), which compares the sequences of samples submitted to the editing protocol with that of a control and estimates the percentage of indels generated at the site of possible cleavage. The higher the percentage of indels, the greater the CRISPR/Cas9 system activity at the site.

### 2.8. Ethics

All experiments were approved by the ethics committee of our institution (Research Ethics Committee—Hospital de Clínicas de Porto Alegre #20160482). Animal procedures were carried out in accordance with the recommendations in the Guide for Care and Use of Laboratory Animals of the National Institutes of Health, monitored by our veterinarian, and designed to minimize animal suffering. Possible gender effects were analyzed in all tests, and no significant differences were found between males and females if not specified.

### 2.9. Statistics

Results were presented as mean ± standard deviation of at least three independent experiments. Group differences were analyzed by Student’s T test or One-Way ANOVA, with Tukey as post hoc, using the PASW Statistics 18 software (v 18.0; SPSS, IBM, Armonk, NY, USA). Group differences in frequency of tumors were calculated through chi square and Fisher’s exact test. Differences were considered statistically significant at *p* < 0.05.

## 3. Results

### 3.1. Physicochemical Characterization of Complexes

The results of physicochemical characterization of complexes are summarized in Table 1. LC exhibited a droplet size of approximately 136 nm, while PDI was below 0.15, indicating monomodal distribution of vesicles. In addition, there was no difference inter days when comparing vesicle diameter, although PDI results increased with storage time. ζ-potential was positive, around +43 mV.

**Figure 1:**
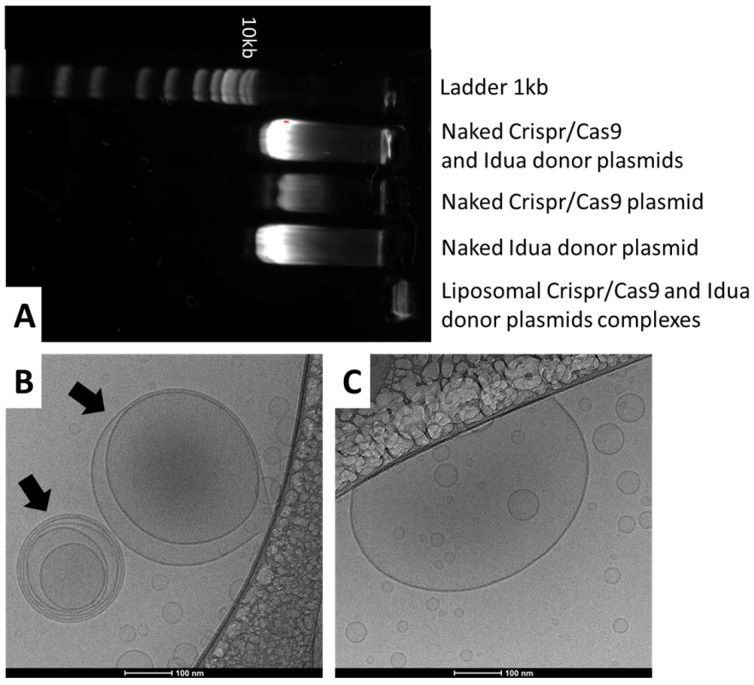
Complexation ability and morphology of LC. (**A**) Gel agarose electrophoresis of naked plasmids and LC. (**B**) Cryo-TEM images showing liposomal CRISPR/Cas9 complexes (LC). Two oligolamellar liposomal complexes can be seen, as pointed to by the arrows. (**C**) A larger LC complex and smaller vesicles. The outer areas are made of supporting carbon film. Scale bars represent 100 nm.

Figure 1A shows the complexation ability of the cationic liposomes in associating the plasmids. As can be seen, when the complexes were electrophoresed, there was no band of running DNA on the gel. In addition, Cryo-TEM images (Figure 1B,C) of the liposomal CRISPR/Cas9 and donor plasmid complexes showed some oligolamellar vesicles, but small-sized vesicles are predominant.

The heterogeneity observed in the size of liposomal vesicles can be attributed to several factors inherent in the complexation process with DNA plasmids. Furthermore, liposome formation occurs in a stochastic manner, being influenced by parameters such as the cationic lipid/DNA ratio, the preparation method, and the electrostatic charge of the components. These factors can result in the generation of vesicles with different sizes and numbers of lipid bilayers, including unilamellar and oligolamellar structures. In addition, the presence of plasmids can induce variations in the curvature of the bilayer, promoting the formation of multillamelar vesicles.

### 3.2. Vector Biodistribution After Intravenous Injection

**Figure 2:**
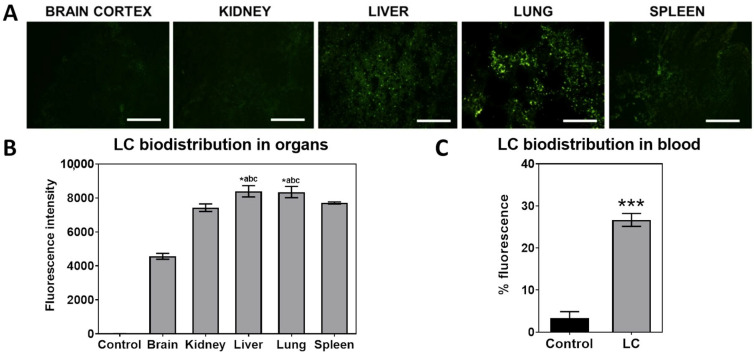
Immediate biodistribution of fluorescent liposomal complexes after injection. (**A**) Representative images of the immediate biodistribution of fluorescent LC complexes in newborn MPS I mice after a single injection in the superficial temporal vein of newborn mice was analyzed under a fluorescence microscope. (**B**) Fluorescence intensity of images (n = 4 treated and 3 untreated controls). (**C**) CL immediate biodistribution in blood measured through flow cytometry (n = 4 treated and 3 untreated controls), *** *p* < 0.001. Images were acquired in fluorescence (Ex/Em = 596/619 nm) at 200× (scale bars 100 µm) magnification. Untreated mice showed no detectable fluorescence in any of the analyzed organs. * abc, *p* < 0.05, significantly different from brain, kidney, and spleen. One-way ANOVA and Kruskal–Wallis post hoc. LC complexes: liposomal CRISPR/Cas9 plasmid and *Idua* donor plasmid complexes.

We observed that our complexes go primarily to the lung and liver, as seen in fluorescence analysis after the immediate biodistribution of complexes (Figure 2). The fluorescent CL also can be found in mice blood, as it is still circulating after 1 min of administration. The number of transgene copies in these organs [7,8], along with kidney, liver, and spleen, made us perform off-target analysis in these organs, as well as on any abnormal-appearing tissues.

### 3.3. Weight and Survival Are Comparable Between Treated and Control Mice Analysis

**Figure 3:**
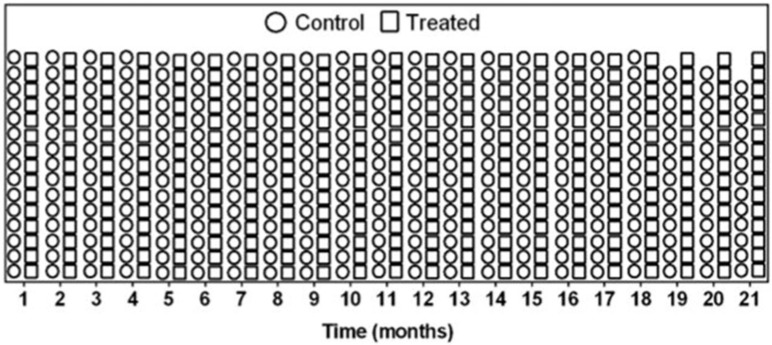
Animal survival during 21 months of experiment. Individual survival of animals from control (dots) and treated (squares) groups. Treatment with LC complexes: liposomal CRISPR/Cas9 plasmid and *Idua* donor plasmid complexes.

Newborn mice were treated and monitored for approximately 21 months to observe long-term effects of the LC treatment. The average body weight of control mice at the end of the experiment was 32.18 ± 3.03 g, while the treated group presented 27.89 ± 3.53 g, showing no statistical difference between groups. The majority of animals survived the 21 months of experiment, besides some of them presenting abnormalities in euthanasia as tumoral mass and organomegaly. Only two animals from the control group were euthanized before the end of the experiment for ethical reasons, at 19 and 20 months, as can be seen in Figure 3. The control group presented an overall rate of survival of 87% at the end of the experiment, while the treated group showed 100% survival.

### 3.4. CRISPR-Cas9-Liposomal Treatment Does Not Increase Tumor Frequency

**Figure 4:**
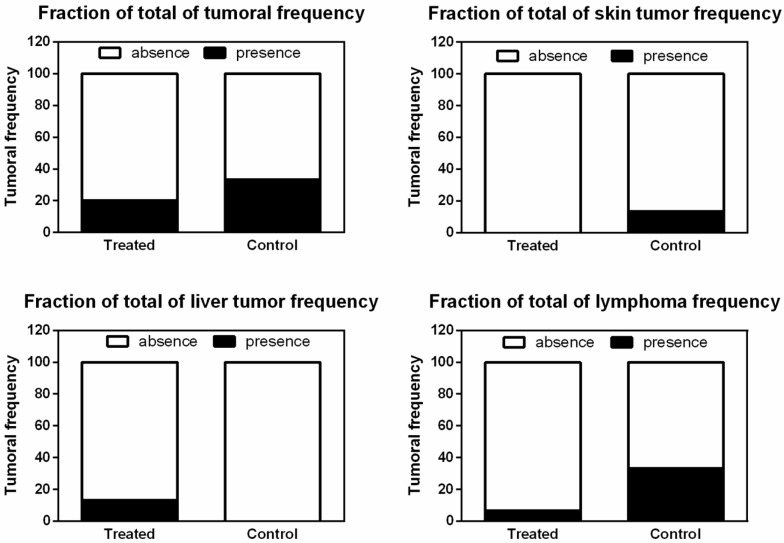
Fraction of total frequency of tumors found in the control (n = 6) and in the treated (n = 6) groups. No significant statistical difference was found when comparing the total frequency between groups (n = 15 per group, chi square test and Fisher’s exact test, *p* < 0.05). Treatment with LC: liposomal CRISPR/Cas9 plasmid and *Idua* donor plasmid complexes.

The overall representations of the analysis of tissues histology and morphology of blood cells are summarized in Figure 4. Newborn mice were treated and monitored for approximately 21 months to observe long-term effects of the LC treatment. There was no statistical difference in frequency of tumors (*p* = 0.6817), skin tumor frequency (*p* = 0.4828), liver tumor frequency (*p* = 0.4828), and lymphoma frequency (*p* = 0.1686) between treated and control groups.

### 3.5. On and Off-Target Analysis

One possible on-target and four possible off-target sites were analyzed in lung and liver samples from treated animals (Table 2). These tissues were chosen because they showed the highest biodistribution of LC [7,8] and the highest percentage of gene editing (about 3%) compared to the other tissues and, therefore, would present a greater chance of occurrence of off-target mutations.

The on-target site is the first one, *Gt(ROSA)26S* or target locus (*Chr6: 113,076,075–113,076,097*). The regions containing the off-target sites were amplified by PCR and sequenced by the Sanger method. The sequences obtained were analyzed by the ICE tool (Synthego, Redwood City, CA, USA), which compares the characteristics of the chromatograms between possibly edited samples and a control, generating an indels score and the possibility of knockouts; therefore, it is possible to evaluate the activity of cleavage and generation of mutations in the analyzed sequences.

**Figure 5:**
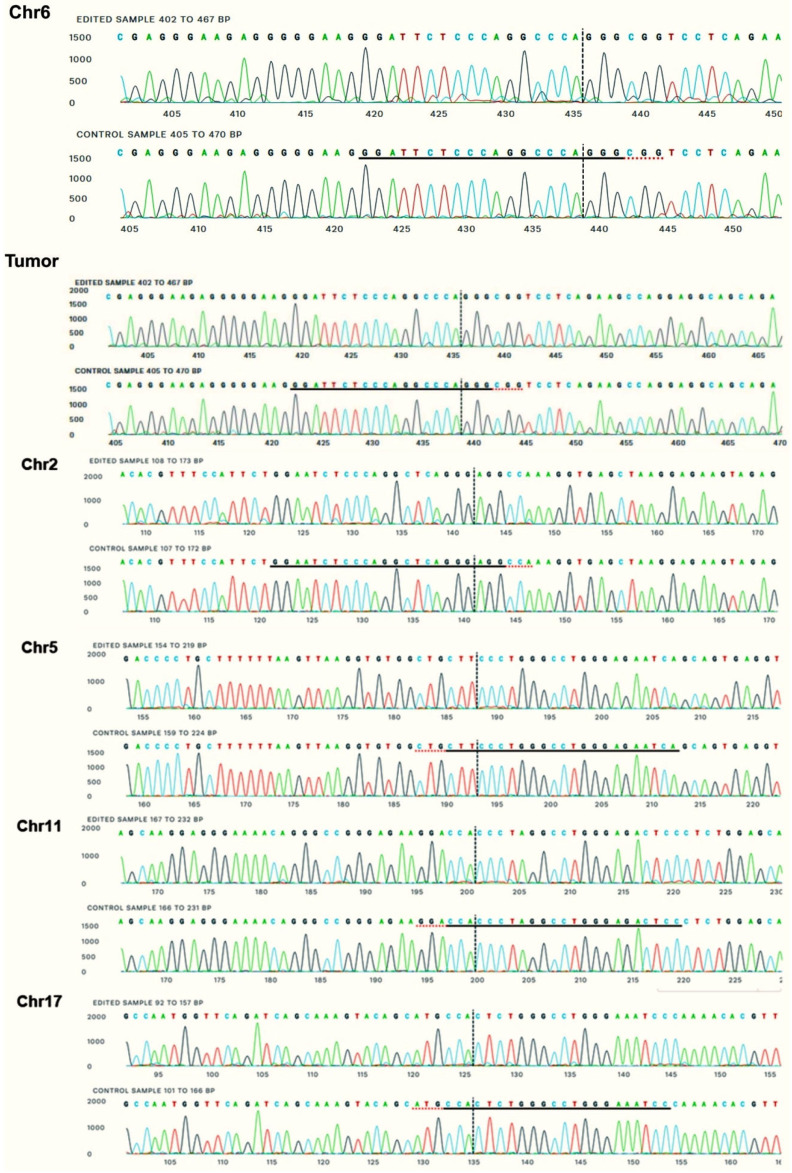
Experimental on and off-target analysis. Example of chromatograms used in the analysis. Each off-target shows sequence variations in relation to the target, because the target is specific and unique in the genome. The red dotted underline represents what the PAM sequence would be; underlined in black is the gRNA sequence. Vertical dots indicate the location where cleavage would occur if the CRISPR/Cas9 system were active at this site.

After the experimental on and off-target analysis, one can observe that there were no signs of alterations in any sample analyzed for all proposed sites, except for the on-target in chromosome 6, which showed 3% transgene copies, corroborating previous studies [7,8]. All possible off-target sites showed 0% indels, as well as the tumor tissue analyzed, removed from a treated animal, with a sequence similarity score of 0.99–1 (maximum), as can be seen in Figure 5.

## 4. Discussion

In this study, we evaluated the safety and the possibility of tumorigenicity after hydrodynamic administration of the CRISPR/Cas9 system and the *Idua* gene donor plasmids complexed to liposomal vectors in newborn mice and their potential to produce off-target effects.

The physicochemical properties of the LC demonstrated the stability of the formulations, with small (around 140 nm) and monomodal (PDI < 0.15) vesicles, even when associated with the plasmids, and morphology corroborates these findings. These characteristics are expected and desirable, as they enhance the likelihood of penetration into target cells. This stability is achieved through the microfluidization procedure and the presence of PEGylated phospholipids, which prevent aggregation and result in small-sized nanostructures [7,8,12]. The ability of the cationic liposome complexation with nucleic acids brings unique morphology and stability to the formulation. These complexes play important roles in transfection and consequent gene editing efficiency [12]. The overall physicochemical characterization of LC is consistent with previous findings from our research group, showing similar particle sizes, charge, and nucleic acids complexation, resulting in stable complexes [4,7,8,12].

The biodistribution of fluorescently labeled LC demonstrated a high affinity primarily for the lungs and liver, corroborating previous studies [7,8]. Efficient delivery to hepatocytes by hydrodynamic injection forces the permeability of the plasma membrane, allowing the complexes to enter the cells [13].

The pulmonary accumulation of cationic liposomes has also been reported [7,14,15], as the fenestrated capillary bed can trap the complexes, inducing effective gene expression in this organ [15]. In addition, low accumulation of LC in the kidneys and spleen can be attributed primarily to their cationic surface charge and bilayered structure, which results in high accumulation and entrapment by the lungs and liver. This can be associated with the fact that nanoparticles smaller than 200 nm tend to avoid retention in the spleen, while larger particles are prone to splenic capture [16,17].

Hydrodynamic injection represents an efficient approach for gene delivery, substantially differing from conventional intravenous injection in both mechanism and transfection efficiency. Furthermore, by bypassing the use of viral vectors, hydrodynamic injection eliminates risks associated with immunogenicity and genomic integration, which is a critical advantage in non-clinical settings and repeated gene therapy applications, especially for delivering naked nucleic acids [13]. DNA biodistribution after hydrodynamic injection exhibits a markedly distinct profile compared to conventional intravenous delivery approaches. Due to the rapid infusion rate and large volume, there is a swift increase in hydrostatic pressure within central blood vessels, particularly the inferior vena cava, which induces a temporary opening of endothelial junctions and facilitates the extravasation of plasmid DNA directly into parenchymal tissues [18,19]. This phenomenon is especially prominent in the liver, which becomes the main target organ of this technique due to its high vascularization [20]. The clinical applicability of hydrodynamic administration still faces significant barriers. Originally developed in murine models, the technique relies on the rapid administration of fluid volumes equivalent to approximately 8–10% of total body volume, which would be impractical and potentially hazardous in humans. However, it stills remain a valid proof of concept for efficient gene delivery to the liver in non-clinical studies [13].

Given the previously described risks of mutagenesis and cancer development, we conducted an analysis of tumor frequency. Comparing the treated and control groups, we found no statistically significant difference in weight, survival, or tumor frequency, suggesting that there is no association between the treatment and an increased risk of cancer. However, animals in both groups developed neoplasms, and unfortunately, two control animals were euthanized before completing 21 months. The presence of these tumors may be associated with common spontaneous tumors that develop in C57BL/6 mouse colonies. A study of mice aged 6 to 18 months of the same strain revealed that sarcomas were the largest category of malignant tumors, followed by hematologic and skin malignancies [21]. A database summary of spontaneous tumor records also showed that the liver, skin, and blood exhibited low frequencies of spontaneous tumors, while leukocytes had a moderate frequency [22]. These findings corroborate the results of our study, where skin, liver, and lymphomas were the most frequently observed tumors.

Although an ideal engineered nuclease would have singular genome-wide specificity, many studies demonstrated off-target events when using CRISPR/Cas9 gene editing tools [23,24]. Multiple mismatches between the guide RNA and its complementary target sequence can be tolerated depending on the quantity, position, and base identity of mismatches, leading to potential off-target events [25]. An off-target event can be defined as a programmable nuclease-induced DNA cleavage at a site anywhere in the genome other than the intended on-target site. Usually, off-target sites are similar in sequence to the desired target sites. However, they may present up to seven mismatches; small indels that cause DNA or RNA bulges; or even a different PAM sequence [26].

When an off-target cutting event occurs, it can be repaired via the non-homologous end joining (NHEJ) pathway, which is inherently error-prone. This often results in small insertions or deletions (indels) at the break site. If these indels cause a frameshift mutation, there can be a loss of gene function due to the production of truncated polypeptides and/or nonsense-mediated mRNA decay [26]. Furthermore, if an off-target cutting event occurs simultaneously with a second cut, it can lead to chromosomal rearrangements such as inversions, translocations, or large deletions between the two break points [27]. These genomic rearrangements can result in loss of heterozygosity (LOH), which is a serious safety concern. Studies have reported that pre-implantation human embryos also utilize the homology-directed repair (HDR) mechanism, where double-strand breaks (DSBs) are repaired by inter-allelic gene conversion using the wild-type homologous allele as a template. As a result, the DSB locus and the adjacent area become identical to the template DNA, leading to LOH. This can result in the homozygosity of deleterious alleles and diseases in the offspring, as well as erase parent-specific DNA epigenetic modifications, leading to imprinting abnormalities [28]. Other concerns reported in the literature about off-target genotoxicity include gene inactivation and the formation of indels at unintended loci, which can affect cell viability or promote tumorigenesis [29].

Considering that the extent of off-target activity is highly dependent on the gRNA, it is essential to identify potential off-target sites and experimentally examine their effects when using CRISPR/Cas systems [26]. Numerous different tools are available for performing in silico off-target predictions, allowing researchers to choose the one that best suits their needs. In this study, we opted to use the COSMID software due to its ease of use and reliability. We performed off-target analyses on the lung and liver, given the number at least of 3% transgene copies in these organs, as indicated by blood flow cytometry showed a 30% increase in blood cells fluorescence and previous reports [7,8]. Comparing the chromatogram characteristics of the possibly edited samples with those of a control, we found a high similarity index, indicating the absence of cleavage activity; no indels were detected in the analyzed sequences as the tumor in one treated sample. Other studies in animal models have also reported the absence or rare occurrence of off-target events [30,31], highlighting that the *ROSA26* locus is widely used and recognized as the “safe harbor locus” for genetic editing in mammals, aligning with the data obtained in this study.

A study conducted by Han and colleagues [31] emphasized the synergy between the positive predictive frequency of genuine off-target sites identified by the COSMID program and NGS (next-generation sequencing) analysis, providing greater confidence in genome editing investigations. It is important to highlight that there are some limitations in this study related to bioinformatics tools for in silico testing of the CRISPR/Cas9 system’s efficiency, such as genetic variability, as they often rely on a reference genomic sequence [26,32]. Chromosomal rearrangements, which frequently involve interactions between different chromosomes, are not accurately modeled in silico due to the lack of detailed spatial and temporal data on chromosome organization within the cell nucleus [33]. Therefore, studies involving whole-genome sequencing would be crucial to ensure the safety of this approach.

Another limitation pertains to Sanger sequencing, which exhibits low sensitivity for detecting low-frequency variants within heterogeneous cell populations; therefore, studies involving whole-genome sequencing would be crucial to ensure the safety of this approach. Examples of HTGTS (High-Throughput, Genome-wide Translocation Sequencing) complements this analysis by specializing in the detection of chromosomal translocations associated with the simultaneous presence of DSBs in different genomic regions. This method is highly relevant for genotoxicity assessment, as it offers a more accurate estimate of the genomic instability caused by editing events [34,35].

Digenome-seq (Digital Genome-wide Off-target Analysis), in turn, stands out as a powerful tool for the unbiased detection of cleavage sites induced by nucleases in denatured genomic DNA in vitro. The main advantage of this technique is its ability to identify off-target events without relying on cellular processes, eliminating physiological variability and enabling a more direct and genomic-level analysis of Cas9 activity [35]. A methodological perspective for our research group is using a global mapping of DNA breaks induced by CRISPR-Cas9, such as GUIDE-Seq. This technique employs the integration of double-stranded oligodeoxynucleotides to unbiasedly identify cleavage sites across the genome. Studies utilizing GUIDE-Seq have demonstrated significant variability in the number of off-target effects associated with different RNA guides, highlighting that the specific characteristics of each sequence directly influence the efficiency and specificity of genome editing. Furthermore, GUIDE-Seq revealed that traditional prediction methods, such as computational analysis and ChIP-Seq, often underestimate the extent of off-target effects. Therefore, this technique is particularly efficient at detecting off-target events in living cells, providing precise and sensitive mapping even within heterogeneous cell populations [36].

An innovative approach involves light-activated chemical modifications of guide RNAs, as described by Qi and colleagues [37]. This strategy uses light-catalyzed click reactions to introduce functional groups into RNA guides, enabling precise temporal and spatial control of CRISPR-Cas9 activity. By applying this methodology, a “CRISPR-OFF switch” can be created to deactivate the system after the desired editing, significantly reducing side effects. This technique enhances safety by limiting residual system activity and broadens its applicability in complex and dynamic genetic editing models, representing a significant advancement toward safer and more precise edits [37].

The probability of occurrence of tumors and activation of oncogenes causing deleterious effects are risks of gene therapy. When the CRISPR system recognizes sequences similar to the target sequence, cleavages that lead to off-target mutations can occur, which can lead to the malfunction of important genes [37,38,39]. This off-target potential has already been reported in several studies with both Cas9 and Cas12 [39]. As the CRISPR system is composed of gRNAs (guide RNAs) that bind to a target genomic locus, mutations may occasionally occur in unwanted genomic loci, and it is important to identify the presence of mutations outside the genomic on-target site. Several studies demonstrate that CRISPR amplification showed increased insertions and/or deletions (indels) in the target DNA, confirmed by NGS and DNA cleavage assays [39,40], reinforcing how imperative it is to perform safety experiments before its use in clinical therapy.

## 5. Conclusions

This study assessed the safety and potential for tumor induction following hydrodynamic administration of the CRISPR/Cas9 system and the *Idua* gene donor plasmid complexed with liposomal vectors in neonatal mice. Analysis of off-target sites, based on in silico predictions, did not detect indels. These findings suggest that the chosen gRNA sequence has the potential to perform genetic editing at the *ROSA26* locus in mice safely, and the experiments conducted provide promising insights for the use of this tool in clinical studies. Therefore, our future efforts will focus on whole-genome sequencing studies to confirm the safety of CRISPR/Cas9 genetic editing approaches.

## Figures and Tables

**Table 1:** Physicochemical characterization of complexes.

Formulation	Days	Mean Diameter (nm)	PDI	ζ-Potential (mV)
LC	0	133.0 ± 3.29	0.101 ± 0.01	+41.50 ± 0.95
	15	139.0 ± 3.05	0.132 ± 0.02 *	+43.80 ± 1.41
	30	138.9 ± 1.01	0.143 ± 0.01 *	+46.07 ± 0.55

PDI: polydispersity index; LC: Liposomal CRISPR/Cas9 and *Idua* donor plasmids complex. * Significant difference from day 0 (*p* < 0.05).

**Table 2:** Potential on and off-target sites obtained with COSMID tool (https://crispr.bme.gatech.edu/ (20 May 2024)).

Result	Query Type	Mismatch	Ends with RG	Chr Position	Strand	Cut Site	Score
GGATTCTCCCAGGCCCAGGGCGG—hit GGATTCTCCCAGGCCCAGGGNGG—query	No indel	0	Yes	Chr6:113,076,075–113,076,097	+	113,076,091	0
TGATTCTCCCAGGCCCAGGGAAG—hit GGATTCTCCCAGGCCCAGGGNGG—query	No indel	2	Yes	Chr5:113,445,565–113,445,587	-	113,445,571	20.12
GGAATCTCCCAGGCTCAGGGAGG—hit GGATTCTCCCAGGCCCAGGGNGG—query	No indel	2	Yes	Chr2:69,238,534–69,238,556	-	69,238,540	2.07
GGAGTCTCCCAGGCCTAGGGTGG—hit GGATTCTCCCAGGCCCAGGGNGG—query	No indel	2	Yes	Chr11:73,171,299–73,171,321	+	73,171,315	2.47
GATTCTCCCAGGCCCAGGGCGG—hit GATTCTCCCAGGCCCAGGGNGG—query	Del 19, or Del 20	0	Yes	Chr6:113,076,076–113,076,097	+	113,076,091	0.63
GATTCTCCCAGGCCCAGGGAAG—hit GATTCTCCCAGGCCCAGGGNGG—query	Del 19, or Del 20	1	Yes	Chr5:113,445,565–113,445,586	-	113,445,571	20.63
GATTCTCCCAGGCCCAGGGCGG—hit GGTTCTCCCAGGCCCAGGGNGG—query	Del 18	1	Yes	Chr6:113,076,076–113,076,097	+	113,076,091	0.79
GGATTTCCCAGGCCCAGAGTGG—hit GGATTTCCCAGGCCCAGGGNGG—query	Del 15	1	Yes	Chr17:8,602,794–8,602,815	+	8,602,809	5.72
GGATTCTCCCAGGCCCAGGGCG—hit GGATTCTCCCAGGCCCAGGNGG—query	Del 1, or Del 2, or Del 3	1	No	Chr6:113,076,075–113,076,096	+	113,076,090	24.51
GGATTCTCCCAGGCCCAGGGCG—hit GGATTCTCCCAGGCCCAGGGGG—query	Del PAM 3	1	No	Chr6:113,076,075–113,076,096	+	113,076,090	40.51
GGATTCTCCCAGGCCCAGGGCG—hit GGATTCTCCCAGGCCCAGGGNG—query	Del PAM 1, or Del PAM 2	0	No	Chr6:113,076,075–113,076,096	+	113,076,090	20.51
GGGATTCTCCCAGGCCCAGGGCGG—hit GNGATTCTCCCAGGCCCAGGGNGG—query	Ins 19	0	Yes	Chr6:113,076,074–113,076,097	+	113,076,091	0.83
GGGATTCTCCCAGGCCCAGGGCGG—hit GGNATTCTCCCAGGCCCAGGGNGG—query	Ins 18	0	Yes	Chr6:113,076,074–113,076,097	+	113,076,091	0.85
GGGATTCTCCCAGGCCCAGGGCGG—hit GGANTTCTCCCAGGCCCAGGGNGG—query	Ins 17	1	Yes	Chr6:113,076,074–113,076,097	+	113,076,091	1.02
GGATTCTCCCAGGCCCAGGGCGGT—hit GGATTCTCCCAGGCCCAGGGNNGG—query	Ins PAM 2, or Ins PAM 3	1	No	Chr6:113,076,075–113,076,098	+	113,076,092	40.7
GGATTCTCCCAGGCCCAGGGCGGT—hit GGATTCTCCCAGGCCCAGGGNGNG—query	Ins PAM 1	1	No	Chr6:113,076,075–113,076,098	+	113,076,092	40.7

## Data Availability

The original contributions presented in this study are included in the article. Further inquiries can be directed to the corresponding author(s).
